# Supplementary material for: Adaptive Tuning Curve Widths Improve Sample Efficient Learning
Source: Front Comput Neurosci. 2020 Feb 18;14:12. doi: 10.3389/fncom.2020.00012 (PMC7041413; doi:10.3389/fncom.2020.00012)
Supplement: Supplementary file 1 [file Data_Sheet_1.PDF]

Supplementary Material

1 MATHEMATICAL ANALYSIS

1.1 Formal statement of the Theorems

We first restate the theorems from the results section of the main paper in a slightly more formal way. For simplifying the notation in the proofs, we rescale the domain and codomain of the function f. Let $f : [n]^{d_A} \to [n]^{d_B}$, where $[n]$ denotes the set of integers $\{1, \dots, n\}$. This rescaling from $[0, 1]$ to $[1, n]$ is convenient as in this way neuron $i \in [n]$ represents value i. Note that this also rescales the error. An error smaller than ℓ implies an error smaller than ℓ/n in the $f : [0, 1]^{d_A} \to [0, 1]^{d_B}$ case. For completeness we also state the static bump algorithm (theory) formally in Algorithm 1 and restate the dynamic bump algorithm (theory) in Algorithm 2.

Algorithm 1: The static bump algorithm (theory) for learning a Lipschitz function $f : [n]^{d_A} \to [n]^{d_B}$. Hyper-parameters: Lipschitz constant or upper bound on Lipschitz constant C, desired precision ℓ, $c = (d_A + d_B + 1)(2C)^{d_A}(3\frac{\sqrt{d_A}+\sqrt{d_B}}{2})^{d_A+d_B}$.

$\hat{L} = \ell/3$; $k^B = 2\ell/(3(\sqrt{d_A} + \sqrt{d_B}))$; $k^A = k^B/C$; $c_{ij} = 0$ for all $i, j \in A \times B$;

for $M = c(\frac{n}{\ell})^{d_A+d_B} \log n$ *samples* **do**

  $x \in_{u.a.r} A$; $I = Int_{k^A}(x)$; $(J, y) \sim Act(I, P, k^B)$;

  if $L(x, y) \geq \hat{L}$ **then** $c_{ij} = c_{ij} - 1$ for all $(i, j) \in I \times J$;

  else $c_{ij} = c_{ij} + 1$ for all $(i, j) \in I \times J$;

for $(i, j) \in I \times J$ **do**

  if $c_{ij} \leq 0$ **then** $p_{ij} = 0$;;

THEOREM 1 (Static bump width k). *Let $f : [n]^{d_A} \to [\ell, n - \ell]^{d_B}$ be a C-Lipschitz continuous function, where ℓ is the desired precision. Assume that the error feedback $L(x, y) = \|f(x) - y\|_2$ is the Euclidean distance between output y and target output $f(x)$. Then, Algorithm 1 achieves with high probability error smaller than ℓ after $c(\frac{n}{\ell})^{d_A+d_B} \log n$ samples, where c is as stated in Algorithm 1.*

THEOREM 2 (Dynamic bump width k). *Let $c_k > 0$. Let $f : [n]^{d_A} \to [c_k n, (1 - c_k)n]^{d_B}$ be a C-Lipschitz continuous function. Assume that the error feedback $L(x, y) = \|f(x) - y\|_2$ is the Euclidean distance between output y and target output $f(x)$. Then, Algorithm 2 achieves with high probability errors smaller than ℓ after $c_1(\frac{n}{\ell})^{d_A} \log n$ samples, where $c_1 = \frac{c}{2^{d^A}-1}\left(\frac{8C}{c_k}\right)^{d_A}$ and c and c_k are defined in Algorithm 2.*

We remark that the restrictions in Theorem 1 and 2, that f maps not too close to the boundary of $[n]^{d_B}$ is required since the neurons close to the boundary have less neighbouring neurons and thus it

Algorithm 2: The dynamic bump algorithm (theory) for learning a Lipschitz function $f:[n]^{d_A}\to[n]^{d_B}$. Hyper-parameters: Lipschitz constant or upper bound on Lipschitz constant C, desired precision ℓ, $0<c_k\le 2/(3(\sqrt{d_A}+\sqrt{d_B}))$, $c=2^{d_A}(d_A+d_B+1)\cdot Vol(S_{d^B}(2/c_k+\sqrt{d_A}/2+\sqrt{d_B}/2))$, where $Vol(S_d(r))$ denotes the euclidean volume of the d-dimensional ball with radius r.

$\hat{L}_0=n$
while $\hat{L}_s\ge\ell/2$ **do**
  $\hat{L}_s=\hat{L}_{s-1}/2$; $k_s^B=c_k\hat{L}_s$; $k_s^A=k_s^B/C$; $c_{ij}=0$ for all $(i,j)\in A\times B$;
  for $M=c\left(\frac{n}{k_s^A}\right)^{d_A}\log n$ *samples* **do**
    $x\in_{u.a.r} A$; $I=Int_{k_s^A}(x)$; $(J,y)\sim Act(I,P,k_s^B)$;
    if $L(x,y)\ge\hat{L}$ **then** $c_{ij}=c_{ij}-1$ for all $(i,j)\in I\times J$;
    else $c_{ij}=c_{ij}+1$ for all $(i,j)\in I\times J$;
  for $(i,j)\in I\times J$ **do**
    if $c_{ij}\le 0$ **then** $p_{ij}=0$;

is rather unlikely that their surrounding interval receives most synaptic input and is activated. This restriction disappears, if one considers a circular output space that encodes for example the head direction of an animal. Formally, one considers the discrete Torus $\mathbb{T}=\mathbb{Z}/n\mathbb{Z}$, consisting of neurons $\{1,\dots,n\}$, where neuron n is attached as neighbour to neuron 1. The error feedback is defined to be the euclidien distance on $\mathbb{T}$, e.g. neuron n has distance 1 to neuron 1, neuron $n-1$ has distance 2 to neuron 1. Then, using $B=\mathbb{T}^{d_B}$ as output space, removes the restriction that f cannot map to close to the boundary of B. Moreover, using $\mathbb{T}^{d_A}$, as input space improves the bound on the sample efficiency in above Theorems by a 2^{d_A} factor, see Remark 5 and 6.

We also restate the lower bound on the sample efficiency:

THEOREM 3 (Lower Bound). *Let $C>0$. There exist C-Lipschitz functions $f:[n]^{d_A}\to[n]^{d_B}$, such that any Algorithm A, that receives for every sample only a constant number of bits as feedback, requires at least $\Omega((\frac{n}{\ell})^{d_A})$ samples*[1] *to achieve accuracy ℓ.*

1.2 Notation and preliminary lemmas

In the paper, we defined the total synaptic input from bump I to bump J as $s(I,J)=\sum_{(i,j)\in I\times J}X_{ij}$, where the X_{ij} are Bernoulli random variables that are 1 with probability p_{ij}. Intuitively, the distributions $s(I,J)$ and $s(I,J')$ are identical if I has identical synaptic probabilities to J and J', and therefore, the activation probabilities of bump J and bump J' should be the same. But if J and J' overlap, then $s(I,J)$ and $s(I,J')$ are not independent which would complicate the theoretical analysis. To overcome this issue, we slightly change the activation procedure here. We define $s(I,J)$ to be the expected total synaptic input $\sum_{(i,j)\in I\times J}p_{ij}$. Then, given an activation I in population A, we sample the output activation J according to an adapted softmax probability

[1] We use Landau notation $\mathcal{O}(.)$, $\Theta(.)$, $\Omega(.)$, ... to denote the asymptotic behaviour with respect to $n\to\infty$

measure $\Pr(J|I) = \frac{e^{s(I,J)}-1}{\sum_{J\in\mathcal{J}} e^{s(I,J)}-1}$. Note that this probability measure is well defined as long as I has at least one outgoing synaptic probability larger than 0. In addition, it has the properties that if all synapses in $I \times J$ are zero, then $\Pr(J|I) = 0$, and if $s(I, J_1) = s(I, J_2)$ then $\Pr(J_1|I) = \Pr(J_2|I)$.

We now define some notations that are convenient for the analysis.

- Recall that for $x \in [n]^d$, we defined $Int_k(x) = Int_k(x_1) \times \ldots \times Int_k(x_d)$, where $Int_k(x_i) = [\max\{0, x_i - k/2\}, \min\{n, x_i + k/2\}]$.
- An (I, J, x, y)-activation consists of bumps I and J with centers x and y, respectively.
- For an (I, J, x, y)-activation, we define $R_k(x, y)$ to be the set of synapses between bump I and bump J. Formally, we define $R_k(x, y) = Int_{k^A}(x) \times Int_{k^B}(y)$, where k denotes the tuple (k^A, k^B). Note that synapse (i', j') being in $R_k(i, j)$ is equivalent to synapse (i, j) being in $R_k(i', j')$.
- We call $R_k(i, j)$ *full* if it contains $(k^A)^{d_A}(k^B)^{d_B}$ many synapses (i, j) with $p_{ij} = 1/2$ and *empty* if it contains no synapses with $p_{ij} > 0$. Otherwise, we call $R_k(i, j)$ *moderate*.
- Further, for a given input neuron i, we define N_i^{full}, N_i^{mod} and N_i^{empty} as the number of output neurons j for which $R_k(i, j)$ is full, moderate and empty, respectively.
- Define $p(j|i) = \Pr(J|I)$ to be the probability that j is the center of the output bump J conditioned on i being the center neuron of the input bump I.

Our first lemma states some bounds on the values $p(j|i)$ that will be useful for the proof.

LEMMA 4 (Bounds on $p(j|i)$). *The following bounds on the probabilities $p(j|i)$ hold.*

1. *If $R_k(i, j)$ is empty, then $p(j|i) = 0$.*
2. *Consider a fixed $i \in A$. For all j with full $R_k(i, j)$, $p(j|i)$ has the same value $p(j|i) = p_i^{full}$. It holds that $1/N_i^{full} \geq p_i^{full} \geq 1/(N_i^{full} + N_i^{mod})$.*
3. *If $R_k(i, j)$ is moderate, then $p_i^{full} \geq p(j|i) \geq 0$.*

PROOF. Let $I = Int_{k^A}(i)$ and $J = Int_{k^B}(j)$. Recall that $\mathcal{J} = \{Int_{k^B}(j) | j \in B\}$ is the set of possible output activations, and that $p(j|i) = \frac{e^{s(I,J)}-1}{\sum_{J'\in\mathcal{J}} e^{s(I,J')}-1}$. Note that $0 \leq s(I, J) = \sum_{i,j\in I\times J} p_{ij} \leq |I \times J|$, $s(I, J) = 0$ holds if and only if $R_{k_s}(i, j)$ is empty, and $s(I, J) = |I \times J|$ holds if and only if $R_{k_s}(i, j)$ is full. From this, the statements of the lemma follow easily.

1.3 Static bump width k

Let us first outline the proof of Theorem 1, before giving the details of proof later in this section. We call a synapse good, if it is inside and not too close to the boundary of the red area in Figure 1 and bad if it is outside and not to close to the boundary of the red area. Formally, a synapse (i, j) is *good* if $\|f(i) - j\|_2 \leq \hat{L} - \hat{d}k^B$ and *bad* if $\|f(i) - j\|_2 > \hat{L} + \hat{d}k^B$, where $\hat{L}$ is the error feedback threshold and $\hat{d} = \frac{\sqrt{d_A}+\sqrt{d_B}}{2}$. We show that good synapses (i, j) can only receive positive counter updates. The synapse receives at least one counter update if j is not to close to the boundary of B, which implies that $p_{ij} = 1/2$ after execution of the algorithm. For bad synapses (i, j), we show that they only receive negative counter updates, which implies that $p_{ij} = 0$ after execution of the

algorithm. This ensures that the error is at most ℓ. Formally, we show that the following 5 properties hold.

1.Good synapses (i,j), i.e. $\|f(i)-j\|_2 \leq \hat{L} - \hat{d}k^B$, obtain only positive counter updates.
2.Bad synapses (i,j), i.e. $\|f(i)-j\|_2 > \hat{L} + \hat{d}k^B$, obtain only negative counter updates.
3.$p_{ij} = \frac{1}{2}$ for all good synapses with $j \in [k^B, n-k^B]^{d_B}$ after execution of the algorithm.
4.$p_{ij} = 0$ for all bad synapses after execution of the algorithm.
5.The error is at most ℓ after execution of the algorithm.

Property 1: Let (i,j) be such that $\|f(i)-j\|_2 \leq \hat{L} - \hat{d}k^B$, and let (I, J, i', j') be an activation such that synapse (i,j) is active, that is, $(i,j) \in R_k(i',j')$. Note that the diagonal of bumps I and J have length $\sqrt{d_A}k^A$ and $\sqrt{d_B}k^B$, respectively. This implies that $\|j-j'\|_2 \leq \frac{\sqrt{d_B}}{2}k^B$, and the Lipschitz continuouity of f implies that $\|f(i')-f(i)\|_2 \leq \|i-i'\|_2 C \leq \frac{\sqrt{d_A}}{2}k^A C = \frac{\sqrt{d_A}}{2}k^B$, where the last equality follows from the choice of k^A and k^B in Algorithm 1. The triangle inequality implies

$$\begin{aligned}\|f(i')-j'\|_2 &= \|f(i')-f(i)+f(i)-j+j-j'\|_2 \\ &\leq \|f(i')-f(i)\|_2 + \|f(i)-j\|_2 + \|j-j'\|_2 \\ &\leq \frac{\sqrt{d_A}}{2}k^B + (\hat{L} - \hat{d}k^B) + \frac{\sqrt{d_B}}{2}k^B \leq \hat{L}\ .\end{aligned}$$

Therefore, synapses (i,j) is only updated for samples with error feedback $L(x,y) = \|f(i')-j'\|_2$ smaller than $\hat{L}$, which implies that the synapse only receives positive counter updates.

Property 2: This statement follows similarly as the last one. Let (i,j) such that $\|f(i)-j\|_2 > \hat{L} + \hat{d}k^B$, and let (I, J, i', j') be an activation such that synapse (i,j) is active. Then, $(i,j) \in R_k(i',j')$ implies that $\|j-j'\|_2 \leq \frac{\sqrt{d_B}}{2}k^B$ and $\|f(i')-f(i)\|_2 \leq \|i-i'\|_2 C \leq \frac{\sqrt{d_A}}{2}k^B$. The triangle inequality implies

$$\|f(i')-j'\|_2 \geq \|f(i)-j\|_2 - \|f(i')-f(i)\|_2 - \|j-j'\|_2 > \hat{L}\ .$$

It follows that synapse (i,j) receives only negative counter updates.

Property 3: Let (i,j) be a good synapse with $j \in [k^B, n-k^B]^{d_B}$. Property 1 states that c_{ij} always increases when synapse (i,j) is active. Thus, $c_{ij} \geq 1$ holds if the synapse is active at least once during the M samples. In the following, we show that this happens with high probability. Note that during the execution of the algorithm we have $p_{ij} = 1/2$ for all $(i,j) \in A \times B$. Therefore, $R_k(i,j)$ is full for all $i \in [n]^{d_A}$ and $j \in [k/2, n-k/2]^{d_B}$, and Lemma 4 implies that $p(j|i)$ is maximal and equal to p_i^{full} for these values of i and j. Denote by $q(j|i) = \sum_{j' \in Int_{k_s^B}(j)} p(j'|i)$ the probability that output neuron j is active given that i is the center neuron of the input. Then $q(j|i) = (k^B)^{d_B} p_i^{full}$ for $j \in [k^B, n-k^B]^{d_B}$. Note that the probability that i' is the center neuron of the input is $\frac{1}{|A|}$. Thus, the probability that synapse (i,j) is active in a fixed round is

$$\sum_{i' \in Int_{k^A}(i)} \frac{q(j|i')}{|A|} = \sum_{i' \in Int_{k^A}(i)} \frac{(k^B)^{d_B} p_{i'}^{full}}{|A|} \geq \frac{(k^A/2)^{d_A}(k^B)^{d_B}}{|A| \cdot |B|}\ , \tag{S1}$$

where the inequality follows from $p_i^{full} \geq \frac{1}{|B|}$ and $|Int_{k^A}(i)| \geq (k^A/2)^{d_A}$ (this lower bound comes from the case that i is in the corner of the input space A). Using the inequality $1-x \leq e^{-x}$, it follows that the probability that synapse (i,j) does never turn active during the $M = \frac{(d_A+d_B+1)\cdot|B|\cdot|A|}{(k^B)^{d_B}(k^A/2)^{d_A}} \log n$ rounds is smaller than

$$\left(1 - \frac{(k^A/2)^{d_A}(k^B)^{d_B}}{|B|\cdot|A|}\right)^M \leq e^{-(d_A+d_B+1)\log n} = n^{-(d_A+d_B+1)} .$$

Then, a union bound over all $n^{d_A+d_B}$ synapses implies that Property holds 3 with probability $1 - \mathcal{O}(n^{-1})$.

Property 4: Property 2 sates that c_{ij} cannot be increased for bad synapses. Therefore, $c_{ij} \leq 0$ and $p_{ij} = 0$ hold after execution of the algorithm.

Property 5: By Property 4 it follows that $p_{ij} = 0$ for $\|f(i) - j\|_2 > \hat{L} + \hat{d}k^B$. The triangle inequality implies that $R_k(i,j)$ is empty for $\|f(i) - j\|_2 > \hat{L} + 2\hat{d}k^B = \ell$, where the last equality follows from the choice of $\hat{L}$ and k^B in Algorithm 1. Thus, by Lemma 4, $p(j|i) = 0$ if $\|f(i) - j\|_2 > \ell$, which implies that the error is smaller than ℓ.

□

REMARK 5. *We comment on how the proof needs to be adapted in order to proof Theorem 1 for circular input and output spaces. As mentioned before, one defines the input and output space to be $A = \mathbb{T}^{d_A}$ and $B = \mathbb{T}^{d_B}$, respectively, where $\mathbb{T} = \mathbb{Z}/n\mathbb{Z}$ is the discrete one-dimensional torus, consisting of neurons $\{1,\ldots,n\}$. We use the euclidean distance $d_{\mathbb{T}}$ on the torus as distance measure and redefine $Int_k(i)$ to be the set of neurons j in $\mathbb{T}$ with $d_{\mathbb{T}}(x,y) \leq k$. Then, the restriction in Property 3 that j should not be close to the boundary of B disappears, because $R_k(i,j)$ is full for all $i \in A$ and $j \in B$. Further, since $|Int_{k^A}(i)| = (k^A)^{d_A}$ for all $i \in A$, the $(1/2)^{d_A}$ factor dissapears in Equation S1, which improves the bound on the number of required samples M by the same factor.*

1.4 Dynamic bump width k

In this section we proof Theorem 2. The proof ideas are analogous to the proof of Theorem 1 in the last section. We will show that similar properties as in the last section hold after each phase of Algorithm 2. In phase s, we call a synapse (i,j) *good* if $\|f(i) - j\|_2 \leq \hat{L}_s - \hat{d}k_s^B$, and we call it *bad* if $\|f(i) - j\|_2 > \hat{L}_s + \hat{d}k_s^B$, where $\hat{L}_s$ is the error feedback threshold of phase s and $\hat{d} = \frac{\sqrt{d_A}+\sqrt{d_B}}{2}$. We show that good synapses (i,j) only receive positive counter updates and that $p_{ij} = 1/2$ holds after phase s if j is not too close to the boundary of B. Note that this boundary condition is more complicated than for the static bump algorithm, because of the iterative application of the static algorithm, c.f. Property 3 below. Bad synapses (i,j) only receive negative counter updates, which implies that $p_{ij} = 0$ after phase s of the algorithm. This implies that the error is at most $2\hat{L}_s$ after phase s of the algorithm. Formally, we show that the following 7 properties hold. We remark that the purpose of Properties 5 and 6 is to facilitate the analysis of the next phase.

1. Good synapses, i.e. $\|f(i) - j\|_2 \leq \hat{L}_s - \hat{d}k_s^B$, receive only positive counter updates during phase s.
2. Bad synapses, i.e. $\|f(i) - j\|_2 > \hat{L}_s + \hat{d}k_s^B$, receive only negative counter updates during phase s.
3. After execution of phase s, $p_{ij} = \frac{1}{2}$ holds for all good synapses (i,j) with $j \in [\sum_{t=1}^{s} k_t^B, n - \sum_{t=1}^{s} k_t^B]^{d_B}$.

4.After execution of phase s, $p_{ij}=0$ holds for all bad synapses.
5.If $\|f(i)-j\|_2 > \hat{L}_s + \hat{d}k_s^B + \hat{d}k_{s+1}^B$, then $R_{k_{s+1}}(i,j)$ is empty after execution of phase s.
6.Let $\alpha > 0$. If $\|f(i)-j\|_2 \leq \hat{L}_s - \hat{d}k_s^B - \alpha\hat{d}k_{s+1}^B$ and $j \in [\sum_{t=1}^{s} k_t^B + \frac{\alpha}{2}k_{s+1}^B, n - \sum_{t=1}^{s} k_t^B - \frac{\alpha}{2}k_{s+1}^B]^{d_B}$ then $R_{\alpha k_{s+1}}(i,j)$ is full after execution of phase s.
7.The error is at most $2\hat{L}_s$ after execution of phase s.

Let us now show how these properties imply the Theorem. Recall that $\hat{L}_s = \frac{n}{2^s}$, $k_s^A = \frac{c_k n}{2^s C}$ and that phase s requires $M_s = c(\frac{n}{k_s^A})^{d_A} = c(\frac{C2^s}{c_k})^{d_A}$ many samples. Property 7 implies that the algorithm reaches error smaller than ℓ in the phase $T = \lceil \log(2n/\ell) \rceil$ for which $\frac{\ell}{2} \leq 2\hat{L}_T = \frac{2n}{2^T} \leq \ell$ holds, which is equivalent to $\frac{2n}{\ell} \leq 2^T \leq \frac{4n}{\ell}$. Therefore, the total number of samples required by the algorithm is

$$\sum_{s=1}^{T} M_s = \sum_{s=1}^{T} c\left(\frac{C2^s}{c_k}\right)^{d_A} = c\left(\frac{C}{c_k}\right)^{d_A} \frac{2^{(T+1)d_A}-1}{2^{d_A}-1} \leq \frac{c}{2^{d^A}-1}\left(\frac{8Cn}{\ell c_k}\right)^{d_A} ,$$

where we used the formula for a sum of a geometric series. This implies Theorem 2.

It remains to prove Properties 1-7. The proof follows by induction over s. Observe that before phase 1 (i.e. $s=0$, $L_0=n$, $k_0^B = c_k n$) all properties are satisfied. Given that all properties are satisfied after phase $s-1$, we show in the induction step that all properties are satisfied with probability $1-\mathcal{O}(n^{-1})$ after phase s. It follows that after phase $T = \lceil \log \frac{2n}{\ell} \rceil$ all properties are satisfied with probability $1-\mathcal{O}(\frac{\log n}{n})$.

Property 1: This statement follows analogously to Property 1 in Section 1.3 by the Lipschitz continuity of f and triangle inequality.

Property 2: The proof is analogous to the proof of Property 2 in Section 1.3.

Property 3: Similar to the proof of Property 3 in the last section, we prove that that $c_{ij} \geq 1$ holds with probability $1-\mathcal{O}(n^{-(d_A+d_B+1)})$ for such synapses. Then, by union bound over all synapses satisfying the assumptions of 3, it holds with probability $1-\mathcal{O}(n^{-1})$ that $c_{ij} \geq 1$ for all these synapses.

Let (i,j) be a synapse satisfying the assumptions of Property 3

$$\|f(i)-j\|_2 < \hat{L}_s - \hat{d}k_s^B \text{ , and} \tag{S2}$$

$$j \in [\sum_{t=1}^{s} k_t^B, n - \sum_{t=1}^{s} k_t^B]^{d_B} \ . \tag{S3}$$

The choice of c_k in the algorithm implies that that $\hat{L}_s - \hat{d}k_s^B \leq \hat{L}_s - \hat{d}k_s^B + \hat{L}_s - 3\hat{d}k_s^B = \hat{L}_{s-1} - \hat{d}k_{s-1}^B - 2\hat{d}k_s^B$. Then, (S3) and Property 6 for phase $s-1$ with $\alpha=2$ imply that $R_{2k_s^B}(i,j)$ is full. Therefore, $R_{k_s}(i',j')$ is full for $i' \in Int_{k_s^A}(i)$ and $j' \in Int_{k_s^B}(j)$, and Lemma 4 implies that $p(j'|i') = p_{i'}^{full}$.

By Property 1, good synapses can only receive positive counter updates. Thus, $c_{ij} \geq 1$ holds if (i,j) is active at least once during the M rounds. Denote by $q(j|i) = \sum_{j' \in Int_{k_s^B}(j)} p(j'|i)$ the probability that output neuron j is active given that i is the center neuron of the input. Note that the probability that i' is the center neuron of the input is $\frac{1}{|A|}$. Thus, the probability that synapse

(i, j) is active for a fixed sample is

$$\sum_{i' \in Int_{k_s^A}(i)} \frac{q(j|i')}{|A|} = \sum_{i' \in Int_{k_s^A}(i)} \sum_{j' \in Int_{k_s^B}(j)} \frac{p(j'|i')}{|A|} = \sum_{i' \in Int_{k_s^A}(i)} \frac{|Int_{k_s^B}(j)| p_{i'}^{full}}{|A|} \ . \tag{S4}$$

Property 5 from phase $s-1$ implies that if $R_{k_s}(i, j)$ is full or moderate, then j lies in the ball with center $f(i)$ and radius $\hat{L}_{s-1} + \hat{d}k_{s-1}^B + \hat{d}k_s^B$. By Lemma 4, it follows

$$p_{i_0}^{full} \geq \frac{1}{N_i^{full} + N_i^{mod}} \geq \frac{1}{Vol(S_{d^B}(\hat{L}_{s-1} + \hat{d}k_{s-1}^B + \hat{d}k_s^B))} \ ,$$

where $Vol(S_{d^B}(r))$ denotes the euclidean volume of the d_B dimensional ball with radius r. Note that $|Int_{k_s^A}(i)| \geq (k_s^A/2)^{d_A}$ (this lower bound comes from the case where i is in the corner of input space A), $|Int_{k_s^B}(j)| = (k_s^B)^{d_B}$, $\hat{L}_{s-1} + \hat{d}k_{s-1}^B + \hat{d}k_s^B = (2/c_k + 3\hat{d})k_s^B$ and $Vol(S_{d^B}((2/c_k + 3\hat{d})k_s^B) = Vol(S_{d^B}(2/c_k + 3\hat{d})) \cdot (k_s^B)^{d_B}$. Thus, the probability from Equation (S4) that synapse (i, j) is active for a fixed sample is at least

$$\sum_{i' \in Int_{k_s^A}(i)} \frac{|Int_{k_s^B}(j)| p_{i'}^{full}}{|A|} \geq \frac{(k_s^A/2)^{d_A}}{Vol(S_{d^B}((2/c_k + 3\hat{d}))) \cdot |A|} \tag{S5}$$

By the inequality $1 - x \leq e^{-x}$, it follows that the probability that synapse (i, j) does never turn active during the $M = \frac{(d_A + d_B + 1) \cdot Vol(S_{d^B}((2/c_k + 3\hat{d}))) \cdot |A|}{(k_s^A/2)^{d_A}} \log n$ rounds is smaller than

$$\left(1 - \frac{(k_s^A/2)^{d_A}}{Vol(S_{d^B}((2/c_k + 3\hat{d}))) \cdot |A|}\right)^M \leq e^{-(d_A + d_B + 1)\log n} = n^{-(d_A + d_B + 1)}$$

which proves the claim.

Property 4: For bad synapses (i, j), Property 2 states that c_{ij} can only be decreased in phase s. This implies that $c_{ij} \leq 0$ and $p_{ij} = 0$ after phase s.

Property 5: For $(i', j') \in R_{k_{s+1}}(i, j)$ the Lipschitz continuity of f, the assumption $\|f(i) - j\|_2 > \hat{L}_s + \hat{d}k_s^B + \hat{d}k_{s+1}^B$ and the triangle inequality implies

$$\begin{aligned} \|f(i') - j'\|_2 &\geq \|f(i) - j\|_2 - \|f(i') - f(i)\|_2 - \|j - j'\|_2 \\ &\geq (\hat{L}_s + \hat{d}k_s^B + \hat{d}k_{s+1}^B) - \frac{\sqrt{d_A}}{2} k_{s+1}^B - \frac{\sqrt{d_B}}{2} k_{s+1}^B \geq \hat{L}_s + \hat{d}k_s^B \ . \end{aligned}$$

The statement follows by Property 4.

Property 6: Let $\alpha > 0$ and (i, j) such that the assumptions are satisfied. Let $(i', j') \in R_{\alpha k_{s+1}}(i, j)$. The Lipschitz continuity and the triangle inequality imply that synapse (i', j') is good:

$$\begin{aligned}\|f(i') - j'\|_2 &\leq \|f(i') - f(i)\|_2 + \|f(i) - j\|_2 + \|j - j'\|_2 \\ &\leq \alpha \frac{\sqrt{d_A}}{2} k^B_{s+1} + \hat{L}_s - \hat{d} k^B_s - \alpha \hat{d} k^B_{s+1} + \alpha \frac{\sqrt{d_B}}{2} k^B_{s+1} \\ &\leq \hat{L}_s - \hat{d} k^B_s \ .\end{aligned}$$

Moreover, $j \in [\sum_{t=1}^{s} k_t^B + \frac{\alpha}{2} k^B_{s+1}, n - \sum_{t=1}^{s} k_t^B - \frac{\alpha}{2} k^B_{s+1}]^{d_B}$ imply that $j' \in [\sum_{t=1}^{s} k_t^B, n - \sum_{t=1}^{s} k_t^B]^{d_B}$. By Property 3, it follows that $R_{\alpha k_s}(i, j)$ is full.

Property 7: By Property 5 and Lemma 4, it follows that $L(x, y) \leq \hat{L}_s + \hat{d} k^B_s + \hat{d} k^B_{s+1} \leq 2\hat{L}_s$, where the last inequality follows from the choice of k^B_s in the algorithm.

□

REMARK 6. *We comment on how above proof needs to be adapted to proof Theorem 2 for circular input and output spaces. When defining input and output space and the error feedback as in Remark 5, then the restrictions on j disappear in Properties 3 and 6. The proofs are easily adapted by ignoring the lines taking care of these restrictions. Further, since $|Int_{k^A_s}(i)| = (k^A_s)^{d_A}$ for all i , the $1/2^{d_A}$ factor in Equation S5 disappears.*

1.5 Lower bound

In this section we prove Theorem 3. Let d_A be the input dimension. Let us construct a Lipschitz function $f : [n]^{d_A} \to [n]$ with Lipschitz constant $C > 0$ for which $(\frac{n}{\ell})^{d_A}$ bits of information are required in order to approximate it with precision ℓ. Let $m = \lceil 2\ell/C \rceil$.Consider the following family $\mathcal{F}$ of Lipschitz functions. For simplicity assume n is divisible by m. For $f \in \mathcal{F}$ it holds that $f(x) = 0$ if $x_i \equiv 0 \mod 2m$ for some i, and $f(x) \in \{-2\ell, 2\ell\}$ if $x_i \in \{m, 3m, 5m, \ldots\}$ for all i. For the points x for which $f(x)$ is not defined yet, we take a linear interpolation between the points defined above. This way the slope of any linear part is at most $2\ell/m \leq C$. Now, let us draw a $f \in \mathcal{F}$ uniformly at random. In order to achieve precision ℓ on the function f, one needs to know for each point on the grid $\{m, 3m, \ldots\}^{d_A}$ whether the function value is 2ℓ or -2ℓ. Therefore, $\Theta((\frac{n}{\ell})^{d_A})$ bits of information are required. Since the algorithm can obtain at most a constant number of bits of information per sample, $\Omega((\frac{n}{\ell})^{d_A}))$ samples are required. If the codomain of the function is multi-dimensional, this lower bound holds as well, by only considering the first output dimension. □

2 HYPER-PARAMETERS FOR EXPERIMENTS

2.1 Sample efficiency comparison (Figure 4)

2.1.1 Dynamic bump algorithm

Table S1 shows the hyper parameters used to obtain the results of the dynamic bump algorithm illustrated in Figure 4 of the main paper. These hyper-parameters were obtained by a coarse grid search. We now give some intuition how the hyper-parameters affect performance of the system.

k output factor: Smaller k output factor yields larger output bumps. Larger output bumps increase the speed of learning, as more synapses are updated, however too large output bumps make learning instable as good synapses may be pruned away, see also Figure S1A.

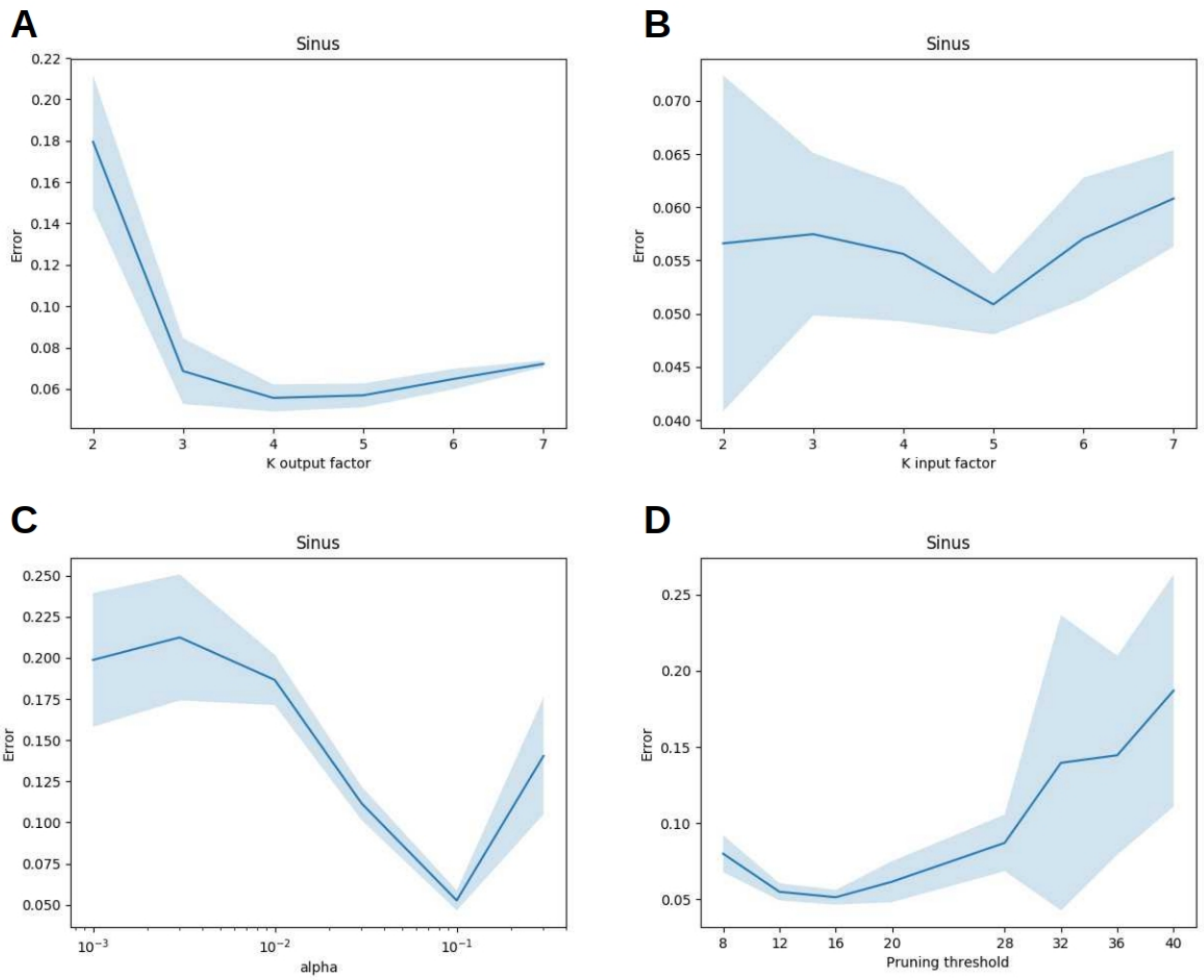

Figure S1: Influence of the hyper-parameters of the dynamic bump algorithm on performance. The hyper-parameters, except the mentioned ones, are set as in Table S1 for the sinus task. (A) c_B is varied and c_A is chosen such that the ratio c_B/c_A is held constant. (B) Only c_A is varied. (C) Only α is varied. Tested α-values are $\{0.001, 0.003, 0.01, 0.03, 0.1, 0.3\}$. (D) Only the pruning threshold θ_{prune} is varied. In all sub-figures, the performance on the sinus task after 1000 training samples is plotted. Mean and standard deviation of 10 runs are shown.

k input factor: Smaller k input factor yields larger input bumps. Larger input bumps cause more synapses to be updated. However, too large input bumps yield unstable learning, because the learning signal is inaccurate for neurons at the borders of the input bump, see also Figure S1B. How inaccurate the learning signal is depends on the slope of the function f. A conservative choice is to choose $k_A = k_B/C$ where C is the Lipschitz constant of f, as done in the theoretical analysis. Consistent to this, both the Lipschitz constant an the optimal k input factor are significantly higher for the polynomial task than for the other tasks.

α (Running mean parameter for the $\hat{L}^i$ updates): Larger α implies that the error thresholds $\hat{L}^i$ adapt faster to the learning progress, however also increases the noise in the $\hat{L}^i$ values. $\alpha = 0.1$ seems to be a good trade off between fast adaptation and noise in the $\hat{L}^i$, see Figure S1C.

Parameters	Sinus	Polynomial	Throw ball	Robotic arm
K output factor c_B	4	4	4	4
K input factor c_A	4	9	4	4
α	0.1	0.1	0.1	0.1
pruning threshold θ_{prune}	12	12	12	12
Minimum number of synapses	5	5	5	5
Initial error threshold $\hat{L}_{init}$	0.5	0.5	0.4	0.4
Input and output layer sizes n	100	100	100	50

Table S1. Hyper-Parameters used for the dynamic bump algorithm in the sample efficiency comparison (Figure 4)

Parameters	Sinus	Polynomial	Throw ball	Reach	Std. par
number of hidden layers	2	2	2	2	2
width of hidden layers	100	100	100	100	100
learning rate	0.1	0.1	0.1	1	0.01
batch size	1	1	1	1	32

Table S2. Hyper-Parameters used for the MLP trained via backprop. in the sample efficiency comparison (Figure 4)

Parameters	Sinus	Polynomial	Throw ball	Reach
number of hidden layers	2	2	2	2
width of hidden layers	10	10	100	100
learning rate	0.1	0.1	0.4	0.3
α	0.03	0.03	0.03	0.01

Table S3. Hyper-Parameters used for the MLP trained via reinforce in the sample efficiency comparison (Figure 4)

Pruning threshold θ_{prune}: It is advantageous to quickly prune away long term inactive synapses to get rid of small areas of strong synapses surrounded by pruned away synapses in the synaptic space. However, θ_{prune} needs to be large enough such that no synapses that do not turn active by chance are pruned away, see Figure S1D. If $\theta_{prune} = 3c_B$ then the probability that this happens is rather low.

Minimum number of synapses: This parameter should be larger than 1. If it is set too large, the algorithm stops before reaching optimal performance.

Initial error threshold $\hat{L}_{init}$: Setting this parameter to small harms performance as good synapses may be pruned away. Setting it too large causes no plasticity in the beginning of training, but does not hamper final performance.

Layer sizes: The layer sizes do not affect performance, as long they are large enough to encode parameters with high enough precision.

2.1.2 Multi-layer perceptron

The hyper-parameters used for the MLPs in Figure 4 are given in Table S2 and Table S3. We used the optimal hyper-parameters found by a grid search for the MLP (backprop, optimal parameters) and MLP (reinforce). For the MLP (std par) we used the suggested default values from Bengio (2012).

Parameters	MountainCarContinuous v0	InvertedPendulum v2
Input grid dimensions	50x50	20x20x20x20
Output grid dimensions	100	50
Policy learning rate	0.1	0.003
Value function learning rate	0.5	0.5
Initial value function	1	140
K input	5	3
K output	5	7

Table S4. Algorithm Hyper-Parameters for the Reinforcement Learning Experiments

2.2 Reinforcement learning experiments (Figure 5)

Table S4 shows the hyper-parameters that where used for the results of the RL-bump algorithm shown in Figure 5. These hyper-parameters where obtained by a coarse grid search over the hyper-parameter space.

REFERENCES

Bengio, Y. (2012). Practical recommendations for gradient-based training of deep architectures. In *Neural networks: Tricks of the trade* (Springer). 437–478
